# 143. Correlation Between Identification of Gram-positive Resistance Genes and Antimicrobial Susceptibility Profiles in Gram Positive Bacteria

**DOI:** 10.1093/ofid/ofad500.216

**Published:** 2023-11-27

**Authors:** Sahrish Ilyas, Rachel Chasan, Michael D Nowak, Gopi Patel, Emilia Mia Sordillo, Meenakshi M Rana, Melissa R Gitman

**Affiliations:** University of Pennsylvania, Philadelphia, Pennsylvania; The Mount Sinai Hospital, New York, New York; The Mount Sinai Hospital, New York, New York; Icahn School of Medicine at Mount Sinai, New York, NY; Icahn School of Medicine at Mount Sinai, New York, NY; Icahn School of Medicine at Mount Sinai, New York, NY; Icahn School of Medicine at Mount Sinai, New York, NY

## Abstract

**Background:**

GenMark ePlex BCID panels detect common gram-positive resistance genes, allowing for earlier optimization of empiric therapy. Data regarding genotypic and phenotypic concordance of isolates remains limited. Our primary objective was to study the prevalence of mecA and mecC among *Staphylococcus aureus* isolates and of vanA and vanB among *Enterococcus spp*. and determine the concordance with phenotypic susceptibility profiles.

**Methods:**

The study was conducted at the clinical microbiology laboratory of Mount Sinai Health System (NY, NY). Samples were collected between March 2019 and April 2021. Presumptive identification and molecular data were obtained from ePlex BCID Panels and compared to phenotypic susceptibility testing performed on Vitek or Microscan systems.

**Results:**

Data were obtained from 1493 isolates tested on the GenMark ePlex BCID gram positive panels. The total prevalence of mecA in *Staphylococcus aureus* isolates was 460/989 = 46.5%. There was no mecC detected. Of the 460 mecA positive *S. aureus* isolates, 99% were oxacillin resistant and 1.0% were oxacillin susceptible. Of the 529 mecA negative *S. aureus* isolates, 98.5% were oxacillin susceptible and 1.5% were oxacillin resistant.

The total prevalence of vanA in enterococcus isolates was 38% (193/504), 81% were detected in E. *fecium sp*, 17% in E. *fecalis sp,* 0.5% in E. *gallinarium sp and* 1.5% in E. *raffinosus sp.* The total prevalence of vanB was 1.1% (6/504). *E. faecium* accounted for 83% and *E. faecalis* for 16% of the isolates. Of the vanA positive isolates, 7/193 (3.6%) were vancomycin susceptible, 2 were e. *fecalis and 5* were e. *fecium* species. None of the isolates in which vanB detected were vancomycin susceptible.

No van gene was detected in 305 of enterococcus isolates. Of these, 250/305 (81%) were *E. faecalis sp* and 36/305 (11%) were *E. faecium sp.* 290/305 total isolates were vancomycin susceptible and 15 were vancomycin resistant, of which 12/15 were *E. gallinarum* and *E. avium* (which are intrinsically resistant), 1 was *E. faecium* and 2 were *E. faecalis*.

**Conclusion:**

Our data shows overall that the susceptibility profile and resistance gene detection by ePlex correlated well with overall minimal discordance. Further studies are needed to determine the optimal management when phenotypic genotypic discordance occurs.

**Disclosures:**

**Meenakshi M. Rana, MD**, Eli Lilly: advisory board

